# Use of the flat panel detector fluoroscope reduces radiation exposure during periacetabular osteotomy

**DOI:** 10.1038/s41598-024-58314-1

**Published:** 2024-04-24

**Authors:** Rachael Martino, Patrick Carry, Jennifer Stickel, Omar Samara, Sterling Lee, Courtney Selberg

**Affiliations:** 1 Children’s Hospital Colorado – Orthopedics Institute, 13123 E 16th Ave, Box 060, Aurora, CO 80045 USA; 2 University of Colorado Anschutz Medical Campus, Aurora, CO USA

**Keywords:** Musculoskeletal system, Medical research, Paediatrics

## Abstract

The Periacetabular Osteotomy is a technically demanding procedure that requires precise intraoperative evaluation of pelvic anatomy. Fluoroscopic images pose a radiation risk to operating room staff, scrubbed personnel, and the patient. Most commonly, a Standard Fluoroscope with an Image Intensifier is used. Our institution recently implemented the novel Fluoroscope with a Flat Panel Detector. The purpose of this study was to compare radiation dosage and accuracy between the two fluoroscopes. A retrospective review of a consecutive series of patients who underwent Periacetabular Osteotomy for symptomatic hip dysplasia was completed. The total radiation exposure dose (mGy) was recorded and compared for each case from the standard fluoroscope (n = 27) and the flat panel detector (n = 26) cohorts. Lateral center edge angle was measured and compared intraoperatively and at the six-week postoperative visit. A total of 53 patients (96% female) with a mean age and BMI of 17.84 (± 6.84) years and 22.66 (± 4.49) kg/m^2^ (standard fluoroscope) and 18.23 (± 4.21) years and 21.99 (± 4.00) kg/m^2^ (flat panel detector) were included. The standard fluoroscope averaged total radiation exposure to be 410.61(± 193.02) mGy, while the flat panel detector averaged 91.12 (± 49.64) mGy (*p* < 0.0001). The average difference (bias) between intraoperative and 6-week postoperative lateral center edge angle measurement was 0.36° (limits of agreement: − 3.19 to 2.47°) for the standard fluoroscope and 0.27° (limits of agreement: − 2.05 to 2.59°) for the flat panel detector cohort. Use of fluoroscopy with flat panel detector technology decreased the total radiation dose exposure intraoperatively and produced an equivalent assessment of intraoperative lateral center edge angle. Decreasing radiation exposure to young patients is imperative to reduce the risk of future comorbidities.

## Introduction

Developmental dysplasia of the hip (DDH) encompasses a wide range of hip joint deformities involving the acetabulum and the proximal femur^1^. DDH can progress into a debilitating condition associated with long-term pain and functional limitations. Periacetabular Osteotomy (PAO) is a widely accepted procedure for the correction of symptomatic hip dysplasia in a skeletally mature individual^2,3^. The PAO has been shown to prevent or delay osteoarthrosis in DDH by changing the mechanics of the joint^4–8^.

The entire pelvis and acetabulum must be clearly visualized during PAO in order to perform precise osteotomies surrounding the acetabulum, properly reorient the acetabular fragment, and perform provision and definitive internal fixation safely. Visualization is done using intraoperative fluoroscopy. Traditionally, a Standard Fluoroscope with an Image Intensifier (SFII) is used. The SFII converts the X-ray beam intensity pattern into an image that is captured by a video camera and has been used since the 1960s^9^. More recently, a Flat Panel Detector (FPD) fluoroscope was implemented at our institution as it more efficiently translates X-ray images to light and turns them directly into electronic data^9,10^. The FPD fluoroscope is insensitive to magnetic fields, has a higher spatial resolution that is independent of its field of view, has no inherent vignetting as well as less geometric “pin cushion” distortion, and a wider dynamic range^9–11^. We decided to start using the FPD and compare it to the SFII for all of the reasons stated above, and for the smaller and more mobile design of the FPD that allows for better control and rotation intraoperatively.

Both the SFII and FPD fluoroscope release ionizing radiation that can be harmful to not only the patient, but also the surgical team and staff^12–15^. Ionizing radiation can cause cellular damage while increasing risks for cancer, especially in the pediatric population^16^. The FPD fluoroscope has been shown to reduce the amount of radiation during central venous catheter placement and reduces the image distortion while increasing navigational accuracy in the pediatric population^10,17,18^, and has been used for dynamic chest X-rays, during a mechanical thrombectomy, and for brachytherapy in the adult population^19–21^. However, the FPD fluoroscope has not been studied in the field of hip preservation.

We sought to find the optimal modality to reduce radiation exposure intraoperatively during PAO. We aimed to determine whether this new technology reduced radiation exposure by comparing the radiation doses between the SFII and FPD fluoroscope. We also compared the accuracy of the FPD fluoroscope relative to the SFII. We hypothesized that the FPD fluoroscope would produce lower levels of radiation and would offer a similar quality image compared to the SFII intraoperatively while performing PAO.

## Materials and methods

Institutional review board approval was obtained from the Colorado Multiple Institutional Review Board (COMIRB) in standard fashion. Due to the retrospective nature of the study, the Colorado Multiple Institutional Review Board waived the need of obtaining informed consent. All methods were carried out in accordance with relevant guidelines and regulations. We retrospectively reviewed a consecutive series of patients who underwent PAO for symptomatic DDH between January of 2019 and August of 2020 at a single institution. All patients had preoperative anteroposterior (AP) pelvis imaging, intraoperative fluoroscopic imaging, as well as a supine AP pelvis radiograph performed at their 6-week postoperative visit. Three patients had a concomitant femoral osteotomy and were excluded.

**Figure 1:**
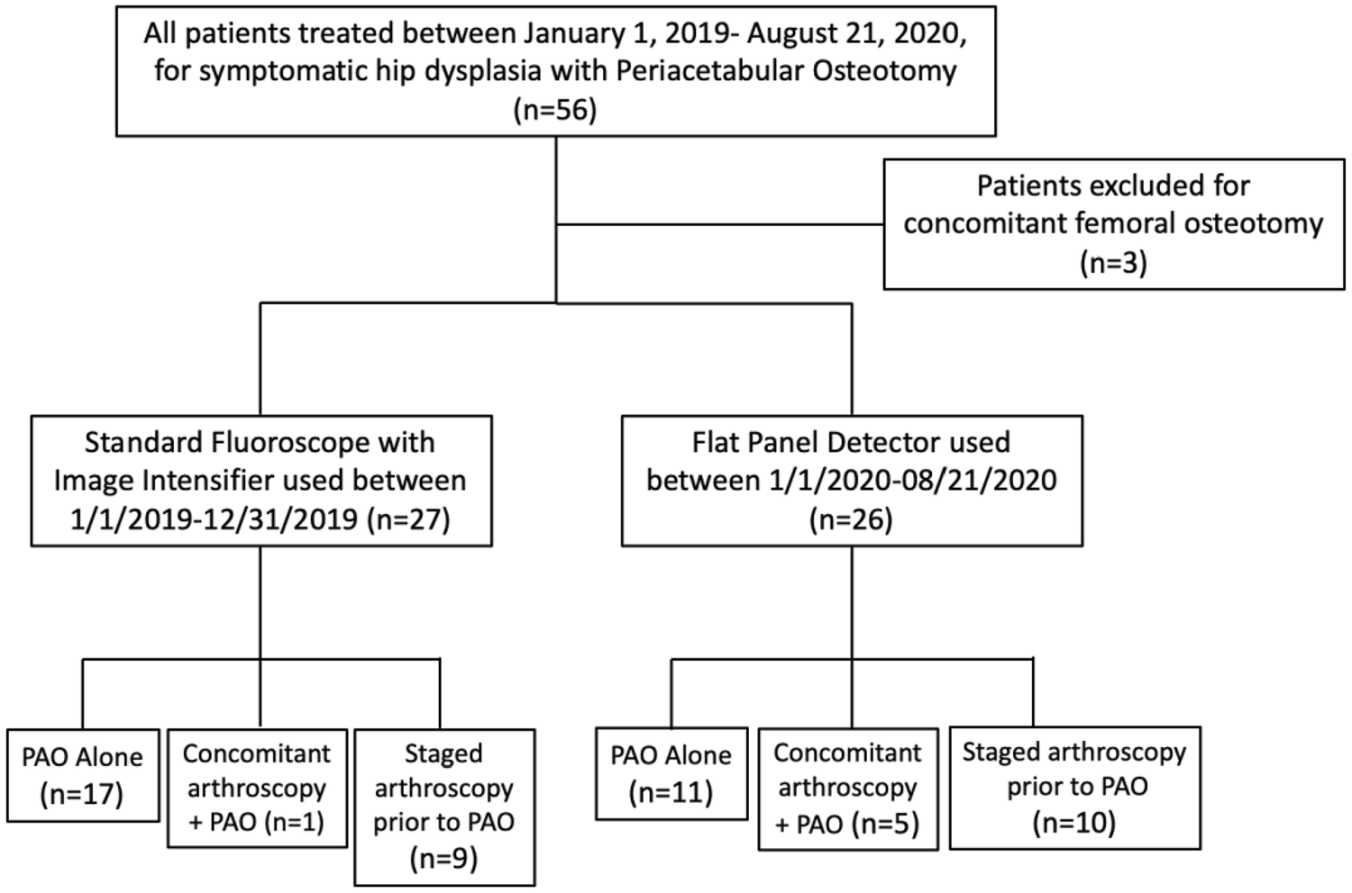
Patient Flow Chart.

We compared two cohorts, those who underwent intraoperative radiographic evaluation using the SFII and those who underwent intraoperative radiographic evaluation using the FPD fluoroscope. The SFII included patients from January of 2019 to December of 2019, while the FPD fluoroscope included those from January of 2020 to August of 2020 (Fig. 1). The SFII is a GE OEC using kV/mA as the imaging technique, while the FPD is a GE OEC Elite also using kV/mA. Demographic variables were collected from the patients’ medical record. The total radiation exposure dose (mGy) was recorded for both cohorts.

**Figure 2:**
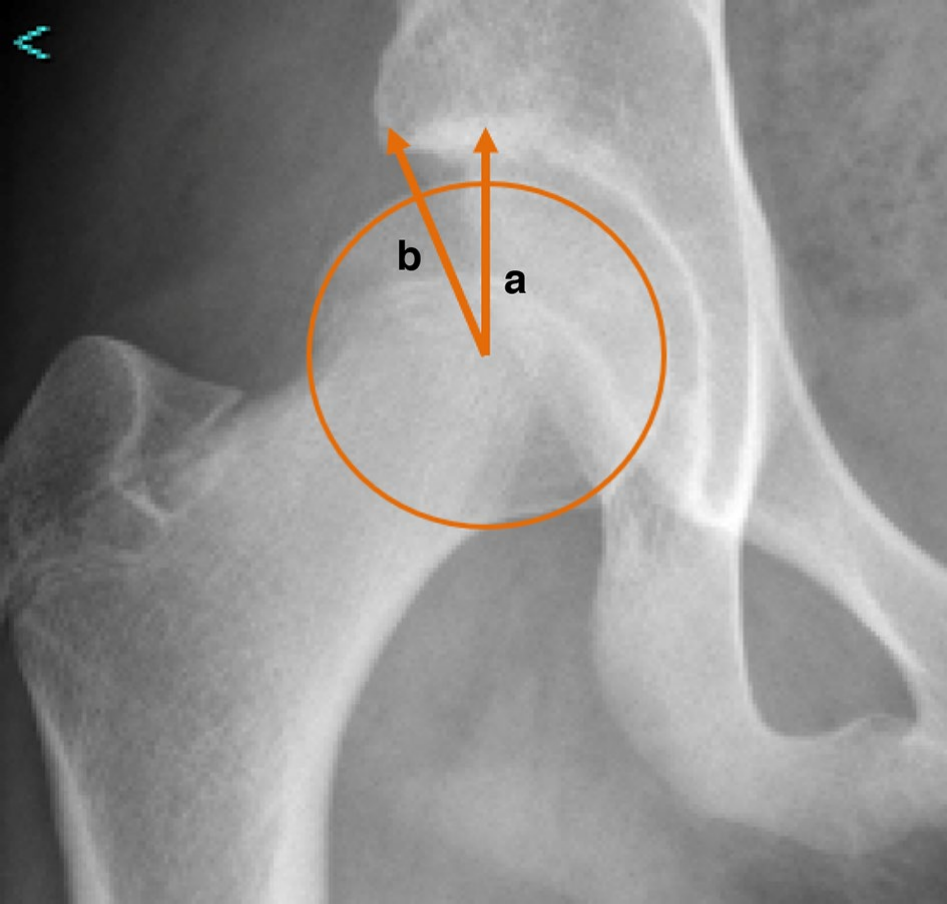
LCEA Measurement. The LCEA is determined by the angle formed between a vertical line through the center of the femoral head that is perpendicular to the transverse axis of the pelvis (**a**) and a line from the center of the femoral head to the lateral acetabular sourcil (**b**).

**Figure 3:**
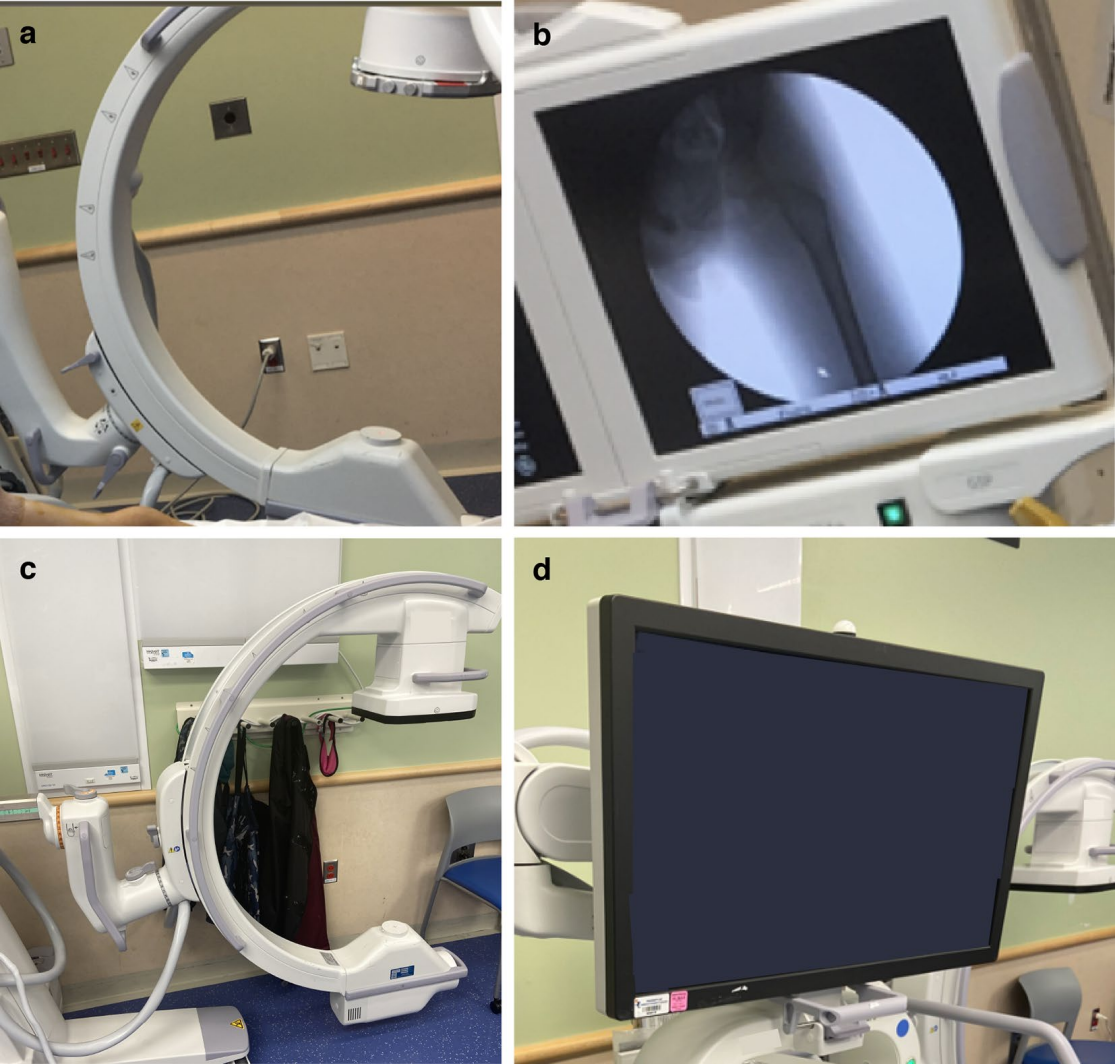
Standard Fluoroscopy with Image Intensifier vs Flat Panel Detector Machines. (**a**, **b**) Standard Fluoroscope with Image Intensifier machine with display. (**c**, **d**) Flat Panel Detector Fluoroscope machine with display.

**Figure 4:**
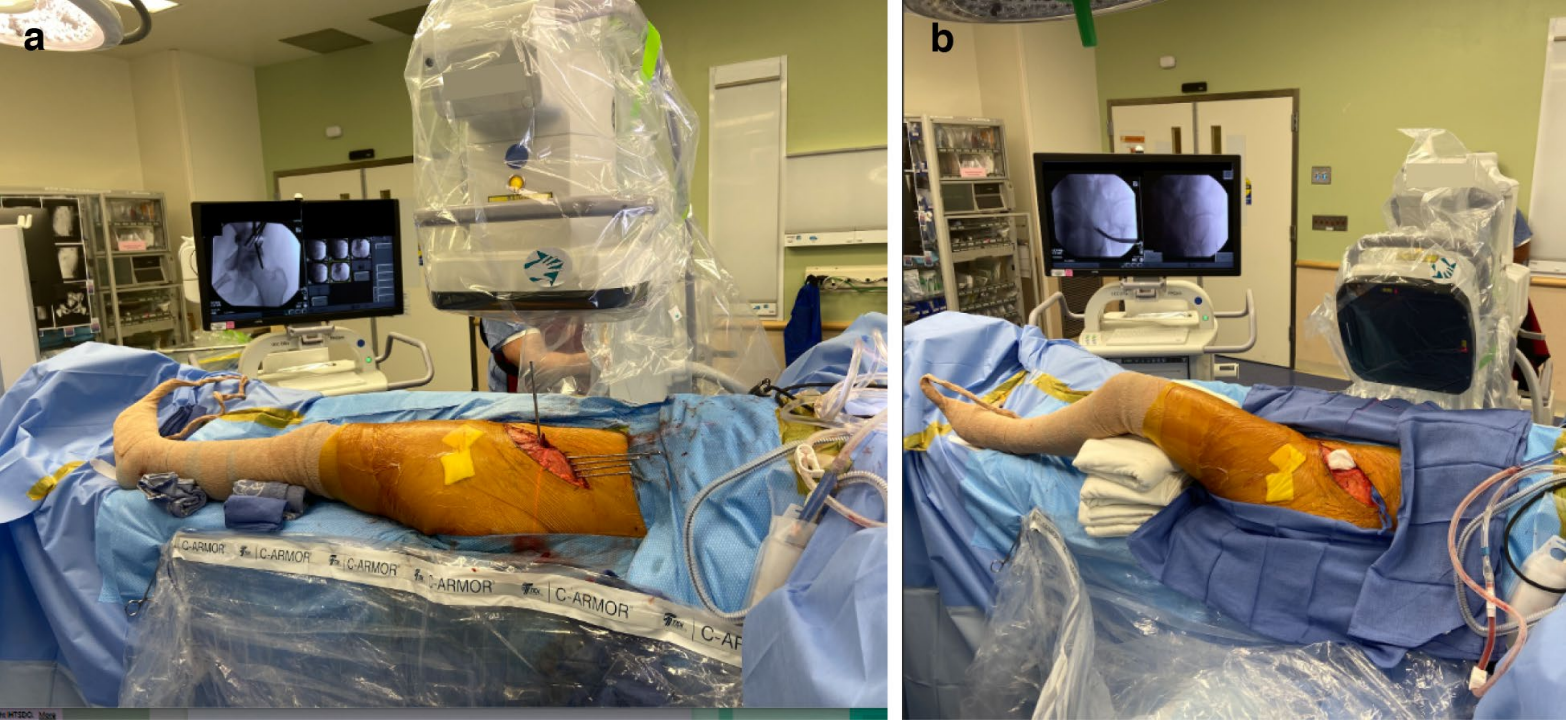
Intraoperative Positioning. Standard intraoperative set up for imaging during PAO using (**a**) AP and (**b**) oblique (faux profil) images.

Lateral Center Edge Angle (LCEA) values were measured in a blinded fashion by a fellowship trained pediatric hip preservation surgeon pre-, intra-, and post-operatively (Fig. 2). LCEA has been determined to be a reliable measure of radiographic correction following PAO with high intra- and inter-observer reliability^22,23^. Intraoperatively, rotation and pelvic tilt were normalized to simulate a true AP pelvis (Figs. 3, 4). The intraoperative LCEA radiographic measures were compared to postoperative LCEA radiographic measures using the supine AP pelvis from the patient’s six-week postoperative follow-up visit.

The two cohorts were also divided into High and Low-risk PAO procedures groups. High-risk patients were identified if they had undergone a previous pelvic osteotomy or anterior approach to the hip for open reduction, had a generalized connective tissue disorder such as Ehlers Danlos Syndrome, or significant medical co-morbidities. We then compared both radiation exposure and LCEA differences intra- and post-operatively within the Low-risk and High-risk PAO groups between the SFII and FPD cohorts.

A radiation physicist was consulted to verify the machines were performing as expected. No performance or dose display issues were identified with either unit during the timeframe of this review. It was verified that both the SFII and FPD machine use the same reference point for the displayed dose. This allows the displayed dose metric to be compared across the studies without the need for mathematical corrections. While the doses reported here are not meant to be patient specific doses, they are used as a quantitative way to compare the doses used in the cases. The dose reference points for both machines are located at the international reference point (IRP). For standard c-arms, this is located 15 cm away from iso-center toward the x-ray tube. It was also found during use that the FPD is a smaller machine and has a better ability to rotate around the operating table.

### Statistical methods

**Figure 5:**
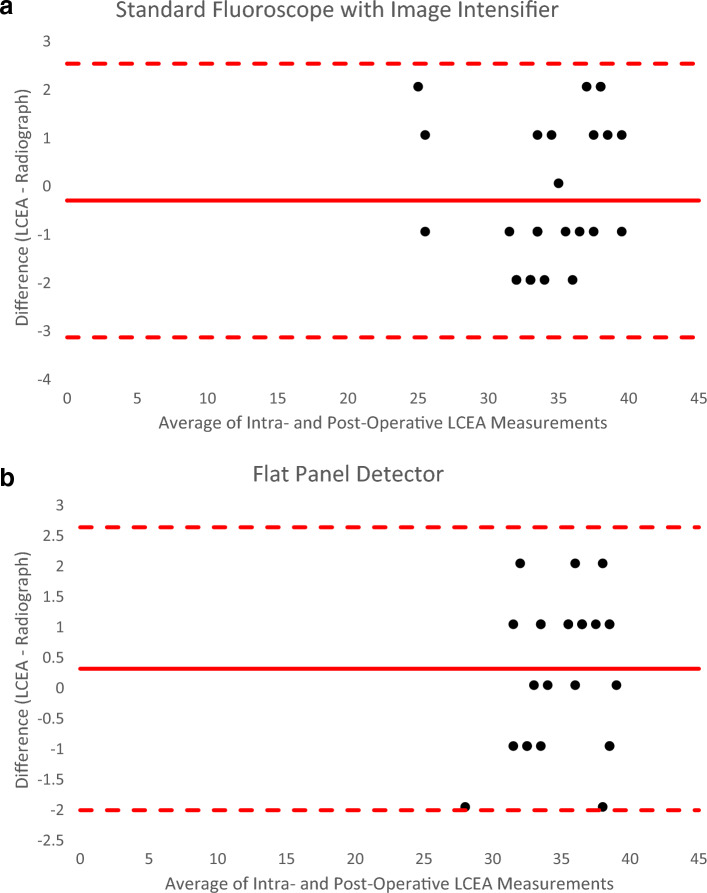
Bland–Altman Plots. (**a**) Standard Fluoroscope with Image Intensifier. (**b**) Flat Panel Detector. Both plots were calculated in degrees.

Descriptive statistics were used to compare the demographic and clinical characteristics in the two cohorts (Table 1). Student’s *t*-test and chi-square tests were used to test for differences in the distribution of clinical characteristics between the two cohorts. Student’s t-test tested for differences in radiation dose between the two cohorts. Bland–Altman methods were used to evaluate the accuracy of the LCEA measurements according to the two radiographic modalities (Fig. 5). Accuracy was calculated as the difference between the intraoperative and six-week postoperative radiographs. The six-week postoperative X-ray measurement was used to represent the gold-standard radiographic measurement. Performance of both radiographic methods relative to this gold-standard were evaluated based on bias, or mean difference, and corresponding 95% limits of agreement. In this analysis, three degrees was considered clinically meaningful^24^.

**Table 1:** Demographics. *At the time of surgery, ^Fisher's exact test, †Chi-Square test

	SFII (n = 27)	FPD (n = 26)	*p*-Value
Average age* (years)	17.84 ± 6.84	18.23 ± 4.21	0.8026
Average BMI* (kg/m^2^)	22.66 ± 4.49	21.99 ± 4.40	0.5836
Gender*^ (% female)	92.59%	100%	0.2369
Ethnicity^ (% Hispanic)	11.11%	11.53%	0.8905
Laterality† (% Right)	55.56%	42.31%	0.2760
Concomitant hip arthroscopy^	1	5	0.0948

## Results

### Demographics

A total of 53 patients were included in this study. There were 27 patients in the SFII cohort and 26 in the FPD cohort. 96% of the total population was female with a mean age of 17.84 ± 6.84 years (SFII) and 18.23 ± 4.21 years (FPD). The average BMI was 22.66 ± 4.49 kg/m^2^ (SFII) and 21.99 ± 4.40 kg/m^2^ (FPD). There were no differences in demographic characteristics between groups (Table 1).

### Radiographic assessment

The average preoperative LCEA was 11.5° in the SFII and the 15.62° in the FPD cohort. There were no differences in the six-week postoperative LCEA measurement in the SFII cohort (34.29° ± 3.84°) compared to the FPD group (35.04° ± 2.78°; *p* = 0.4107; Table 2).

**Table 2:** Comparison of surgical radiation characteristics in the two fluoroscope groups.

	SFII (n = 27)	FPD (n = 26)	*p*-Value
Average radiation dose (mGy)	410.61 ± 193.02	91.12 ± 49.64	< 0.0001
Average exposure time (mGy)	15.15 ± 3.31	6.21 ± 1.08	< 0.0001
Average surgical time (Hr)	9.09 ± 1.44	6.74 ± 0.86	< 0.0001
Average dose/ surgical time (mGy/Hr)	44.25 ± 20.13	13.63 ± 7.57	< 0.0001
Average exposure time/surgical tine (min/Hr)	9.32 ± 1.60	0.93 ± 0.15	< 0.0001

### Radiation exposure

There was a 78% reduction in average total radiation dose in the FPD fluoroscope group, 91.12 mGy, compared to the SFII cohort, 410.61 mGy (*p* = 0.0001). There was also a 59% decrease in radiation exposure time between groups. There was a significant difference between the two cohorts for both radiation exposure dose and radiation exposure time (Table 3). The average radiation dose rate (mGy/second) was statistically different between the two groups. The SFII cohort averaged 0.44 mGy/second, while the FPD cohort averaged 0.24 mGy/second (*p* < 0.0001). This demonstrates the higher efficiency of the FPD machine that can lead to a decreased dose even when the overall fluoro exposure time is not significantly lower. Additionally, the average kV/mA for the SFII was 80.08/2.75 and the FPD was 76.41/0.69.

**Table 3:** LCEA Measurements.

	SFII (n = 27)	FPD (n = 26)	*p*-Value
Average preoperative LCEA	11.5 ± 12.62	15.62 ± 7.36	0.1469
Average intraoperative LCEA	33.93 ± 4.03	35.31 ± 3.06	0.1608
Average postoperative LCEA	34.29 ± 3.84	35.04 ± 2.78	0.4107

The average surgical time was significantly reduced in the FPD cohort, 6.74 ± 0.86 h, relative to the SFII cohort, 9.09 ± 1.44 h (*p* < 0.0001). Therefore, we also compared the average radiation dose per surgical hour between the two cohorts to decrease surgeon experience bias. Consistent with the primary analysis, the average radiation dose per hour of surgical time was significantly less in the FPD cohort, 13.63 mGy/hr ± 7.57, compared to the SFII cohort, 44.25 mGy/hr ± 20.13 (*p* < 0.0001). Each machine also measured the total radiation exposure time per case. We used this value to calculate the average radiation exposure time per surgical hour: 9.32 ± 1.60 min/hr (SFII) and 0.93 ± 0.15 (FPD, *p* < 0.0001), which was also significantly different between groups.

Finally, we compared both the high-risk and low-risk procedures (Table 4). The average radiation dosage for the high-risk procedures were 398.31 mGy (SFII) and 94.19 mGy (FPD, *p* < 0.0001) and were 416.43 mGy (SFII) and 89.00 mGy (FPD) for low-risk procedures (*p* < 0.0001). Finally, the average radiation dosage per exposure time for the high risk patients was 0.44 mGy/second (SFII) and 0.27 mGy/second (FPD, *p* < 0.0001). As for the low risk patients the SFII cohort averaged 0.42 mGy/second, while the FPD cohort averaged 0.22 mGy/second (*p* < 0.0001). The differences between groups were consistent among high versus low-risk patients in the two cohorts (Table 4).

**Table 4:** High vs low risk procedures.

	SFII (n = 27)	FPD (n = 26)	*p*-Value
High risk (HR)	8 (29.6%)	9 (34.6%)	0.6975
Low risk (LR)	19 (70.4%)	18 (69.2%)	0.9280
HR Surgical time (Hr)	9.13 ± 2.09	6.87 ± 0.60	0.0184
LR Surgical time (LR)	9.07 ± 1.13	6.67 ± 0.99	< 0.0001
HR Radiation dose (mGy)	388.67 ± 154.16	94.19 ± 52.01	0.0007
LR Radiation dose (mGy)	416.43 ± 214.85	89.49 ± 49.90	< 0.0001
HR Radiation dose/surgical time (mGy/Hr)	42.25 ± 13.99	13.63 ± 22.54	0.0004
LR Radiation dose/surgical time (mGy/Hr)	45.79 ± 22.54	13.64 ± 8.02	< 0.0001

### Accuracy

The bias, or mean difference, between the intraoperative and 6-week postoperative LCEA measurements was 0.36° for the SFII cohort and 0.27° for the FPD cohort. The limits of agreement were [− 3.19 to 2.47] for the SFII cohort and [− 2.05–2.59] for the FPD cohort.

### Radiation exposure and hip arthroscopy

A subset of patients in our cohort underwent a concomitant hip arthroscopy (Table 1). Total radiation dosage in this subset of patients was difficult to determine because the radiation dosage could not be separated into individual procedures based on the imaging report available and the retrospective nature of the study. Therefore, we conducted a sensitivity analysis to determine whether the inclusion of this subset of patients impacted the observed difference in radiation dose and radiation dose per surgical hour between the two groups. Among individuals who underwent PAO only, the average radiation dosage was significantly (*p* < 0.0001) higher in the SFII group (408.83 mGy) group compared to FPD group (83.14 mGy). Similarly, the average radiation dosage per surgical hour was significantly (*p* < 0.0001) higher in the SFII group (44.37 mGy/hr) compared to the FPD group (12.27 mGy/hr). Overall, after removing patients who underwent a concomitant arthroscopy, the differences radiation dose and radiation dose per surgical hour between groups were consistent with the primary analysis.

## Discussion

Reduction of radiation exposure to young patients and operating room personnel is of the utmost importance while maintaining the quality of surgical correction during PAO surgery. Previous literature has described the amount of radiation an orthopedic surgeon is exposed to intraoperatively. According to Canham et al., during an arthroscopic hip procedure there is an average radiation dosage of 490 mrem (4.9 mGy) to the surgeon^25^. We recognize that the PAO is a longer surgical procedure compared to a hip arthroscopy, but this is the closest comparable dose reporting thus far in the literature. We demonstrate in this single-institution retrospective review that the FPD fluoroscope has the potential to decrease direct radiation, including both exposure time, dose, and dose rate during PAO while maintaining a similar quality of surgical correction as compared to a traditional fluoroscope. Additionally there was a decrease in mA which suggests that the FPD uses less than half the mA compared to the SFII to achieve the same image quality. This explains the lower radiation dosage produced by the FPD due to the higher efficiency of the detectors that allows for fewer x-rays to get the same noise level.

Imaging during PAO requires the pelvis to be level and visualized as similarly to a true AP pelvis image as possible. This allows for an accurate prediction of the patient’s true standing alignment once acetabular reorientation is final and recovery is complete. The FPD fluoroscope allows for a wider field of view to visualize a larger portion of the pelvis in a single image which may allow less fluoroscopic shots to be taken. This decrease in radiation exposure has positive implications for both the patient as well as operating room personnel. Results from this retrospective study demonstrate a significant reduction in radiation during PAO with use of FPD technology compared to the SFII.

The radiation dosage and exposure time are able to be directly compared between the SFII and FPD even though they are different machines because they both use the same reference point. This allows us to compare radiation dosage between patients no matter where the table is located because the reference point, that the dose is calculated from, will always be the same between patients and machines. This is also why we decided to calculate the average radiation dose rate. We can see that there was a significant decrease using the FPD fluoroscope, suggesting this machine significantly decreases radiation exposure compared to the SFII.

**Figure 6:**
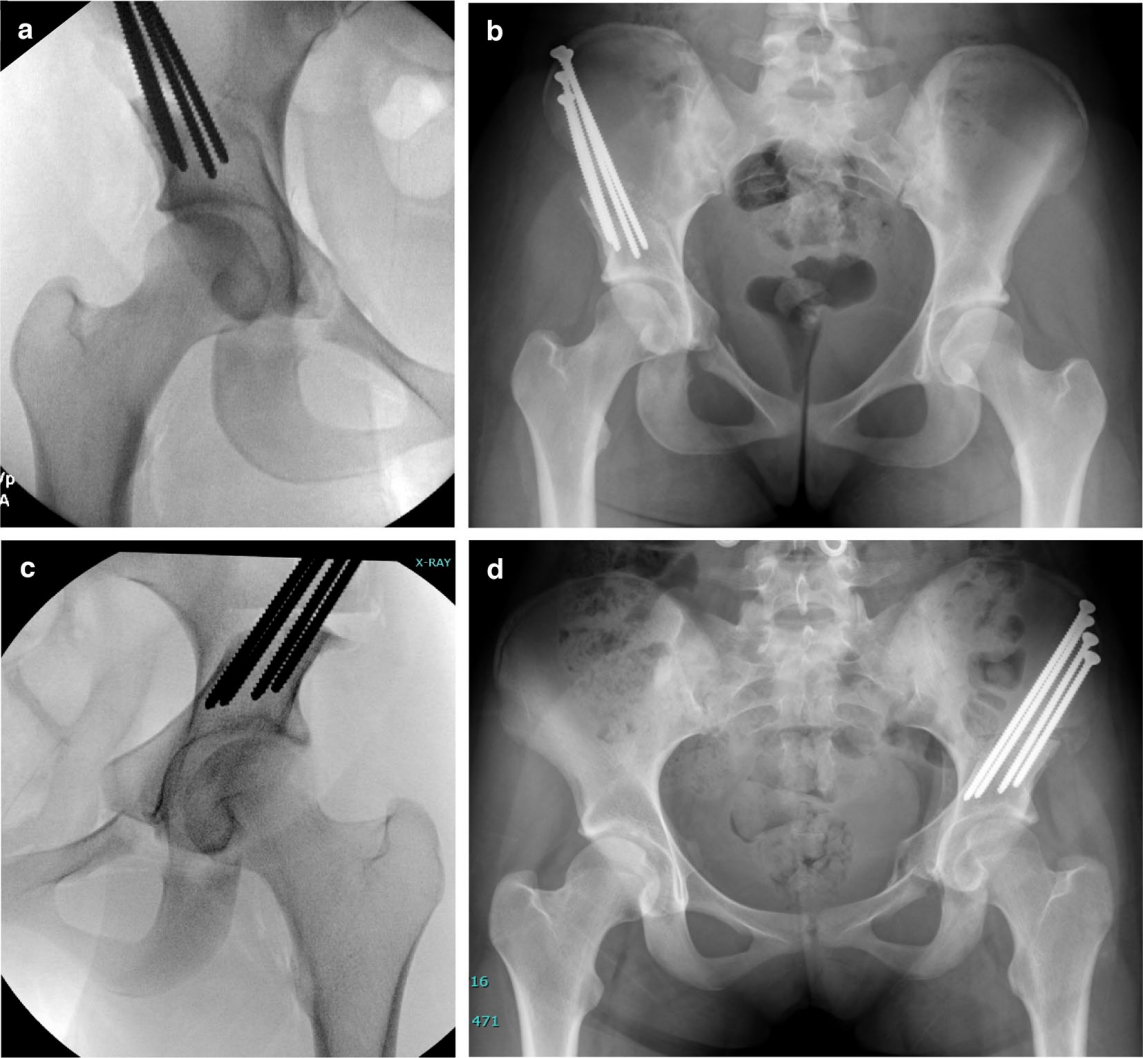
Intraoperative fluoroscopic images compared to their anteroposterior (AP) pelvis radiograph taken 6-weeks postoperatively. (**a**) Patient 1 image taken by the Standard Fluoroscope with an Image Intensifier intraoperatively. (**b**) Patient 1 AP pelvis taken six-weeks postoperatively. (**c**) Patient 2 image taken intraoperatively by the novel Fluoroscope with Flat Panel Detector Fluoroscope. (**d**) Patient 2 AP pelvis taken six-weeks postoperatively.

Use of a novel imaging modality necessitates that the visualization of pelvic anatomy, performance of osteotomies, and acetabular reorientation is possible with the same quality as the previous standard of care. The benefit of reduction in intraoperative radiation exposure during PAO must be accompanied by similar image quality, and the ability to achieve similar results as compared to the SFII. In this case, the bias, or difference between the intraoperative and postoperative radiographs, was low in both groups without a significant difference in LCEA. Importantly, neither machine consistently over- nor under-estimated the intraoperative LCEA as compared to its respective patient’s postoperative LCEA. Additionally, on direct visual estimation, both the SFII and FPD produced similar quality images as observed by viewing the two images side by side (Fig. 6). Overall, these results indicate no significant difference between the image quality produced by the FPD fluoroscope as compared to the SFII.

There were a similar number of patients who underwent concomitant hip arthroscopy during PAO between the two groups (Table 1). The radiation specifically attributable to hip arthroscopy procedure was not able to be ascertained from retrospective review of the electronic medical record. However, there was a significant decrease in radiation dosage and radiation dosage per surgical time in the FPD cohort compared to the SFII when all concomitant arthroscopy patients were removed. Patients in which the procedure was separated by 1–2 weeks also showed a similar trend in decreased radiation exposure within the FPD group.

There were several limitations to this study. All cases were performed by a single surgeon from a single institution in the first five years of the surgeon’s career. The cases were included in a consecutive manner, and there was a significant decrease in surgical time between patients in the SFII cohort which were performed in the initial half of the study time period, versus the FPD group which were performed in the latter half of the study. We acknowledge the presence of a substantial learning curve in regards to PAO surgery, which has been previously reported in the literature^26^. However, when radiation dose was normalized per time, the average radiation dose per surgical hour was still significantly less in the FPD group. Additionally, when comparing high and low-risk procedures we saw a similar decrease in radiation dosage and radiation dosage per surgical hour in the FPD cohort. We also acknowledge that with experience comes the necessity for less fluoroscopic images per surgical case, and we accounted for this by normalizing the radiation dose per surgical hour. There is no record of exact number of fluoroscopic shots performed; however both radiation dosage and total fluoroscopy time were still substantially less in the FPD group even when normalized per hour of surgical time.

This study was also limited by a short duration of follow-up, as comparison was made to 6-week postoperative imaging. We cannot determine the long-term effects the use of the FPD fluoroscope had on this cohort. Further study is necessary to develop an ideal multi-surgeon imaging protocol to reduce radiation exposure to young patients, while still providing adequate image quality and surgical correction.

Finally, this study was limited by evaluation of only LCEA intra- and post-operatively. LCEA was chosen as a quality assessment parameter given its high intra- and inter-observer reliability. Agreement exists in the literature regarding the LCEA as one of the most common metrics to assess appropriate acetabular correction^27^. Many factors have been identified as predictors of poorer survivorship of the native hip following PAO, including increased Tönnis arthritis grade, older age, higher BMI, and female sex^27^. The “acetabular safe zone” of acetabular three-dimensional orientation has been identified, which is a complex task^27^. It remains unclear what the ideal position ought to be for each individual patient to optimize the biomechanical environment of the hip joint^27^. LCEA between 30°and 40° was identified as the range which would optimize outcomes within the 4–12 year follow-up period^28^; and an LCEA of greater than or equal to 32° demonstrated delayed osteoarthritis progression^27,28^. However, both the acetabular version as well as anterior coverage, measured by ACEA, have both been shown to correlate with PAO outcomes; further work will be necessary to examine the differences between SFII and FPD fluoroscopes in regards to these measures. Hip preservation surgeons are still in the process of identifying the ideal target for acetabular reorientation, and whether surgeons can reproducibly hit this target with every patient^27,28^.

It is crucial to optimize the accuracy of intraoperative imaging during PAO surgery while minimizing radiation exposure to staff and the patient. In this retrospective, single-surgeon study, we demonstrate a significant reduction in radiation exposure using the FPD fluoroscope intraoperatively during PAO while maintaining accuracy of the acetabular reorientation based on LCEA. Further work will be necessary to examine if there are differences in these two fluoroscopic techniques in regards to long-term outcomes and other parameters of acetabular reorientation.

## Data Availability

The datasets generated during and/or analyzed during the current study are available from the corresponding author upon reasonable request.
